# Coronary Embolism despite CHA₂DS₂-VASc Score of Zero: Should We Reconsider Anticoagulation?

**DOI:** 10.1155/2021/9912245

**Published:** 2021-07-31

**Authors:** Ammar Ahmed, Andrew Assaf, Aditi Shankar, Marcel Zughaib

**Affiliations:** ^1^Department of Internal Medicine, Ascension Providence Hospital, Southfield, MI, USA; ^2^Department of Cardiovascular Disease, Ascension Providence Hospital, Southfield, MI, USA; ^3^Department of Internal Medicine, Texas Health Presbyterian Dallas, Dallas, TX, USA

## Abstract

Coronary embolism (CE) is a rare but important cause of acute coronary syndrome. The most common source of emboli is considered to be infective endocarditis and atrial fibrillation. Various studies have estimated the prevalence of coronary embolism; however, diagnosis is challenging. Often, it is difficult to differentiate. Nonetheless, this is an important step as treating the underlying cause of an embolism is essential to limit recurrence. However, while this condition may have fatal consequences, due to its uncommon occurrence, there is no consensus on diagnosis and management. We present a case of a 53-year-old obese male, with a history of paroxysmal atrial fibrillation not on anticoagulation due to a low CHA_2_DS_2_-VASc score, who presented with chest pain associated with lightheadedness. ECG on admission revealed coarse atrial fibrillation, and troponin was gradually elevating on serial lab workup. Coronary angiography revealed a distal left anterior descending artery occlusion with apical wall akinesis without any evidence of atherosclerotic coronary artery disease. A presumptive diagnosis of coronary embolism secondary to paroxysmal atrial fibrillation was made, and the patient was started on anticoagulation despite a low CHA_2_DS_2_-VASc score. This case not only highlights coronary embolism but also illustrates that a low CHA_2_DS_2_-VASc score does not mean there is no risk of emboli. For such patients, it is important to take clinical reasoning into account along with the CHA_2_DS_2_-VASc score to determine the benefit of anticoagulation.

## 1. Introduction

Of patients presenting with acute coronary syndrome, a minority will have a coronary embolism (CE) [1]. This is a rare occurrence of nonatherosclerotic coronary artery disease; nonetheless, it can have fatal consequences. Studies conducted in 1978 and 1989 found that 4-7% and 13% of all ACS cases at coronary angiography or autopsy, respectively [2, 3]. The most recent study on prevalence was conducted by Shibata et al. in 2015, who demonstrated 2.9% of patients with ACS were due to CE [4]. The most common sources of emboli are infective endocarditis and nonvalvular atrial fibrillation [4, 5]. Other sources include cardiac tumors, paradoxical emboli through a patent foramen ovale, or iatrogenic [1]. Importantly, Shibata et al. also reported that over 50% of the patients in their study with CE and atrial fibrillation had a CHADS_2_ less than 2 [4]. While some of these patients would have higher scores using the CHA_2_DS_2_-VASc score, it is important to note neither scoring system account for a CE event. Currently, optimal therapy has not been established [4]. Patients with a reversible cause of thromboembolism are recommended to receive 3 months of anticoagulation, in line with the management of venous thromboembolism [1]. However, patients with atrial fibrillation as a source of embolism can have recurrence and should receive continuous anticoagulation even with a low CHA_2_DS_2_-VASc score [1, 4]. This is important as a second CE event can be preventable. Patients who have undergone PCI should also be on antiplatelet therapy [1].

The mechanism of cardiac embolism in atrial fibrillation can be explained by Virchow's triad of endothelial damage, hypercoagulability, and stasis [6, 7]. Due to the anatomy of the left atrial appendage, it constitutes the microenvironment for thrombus formation during and after an episode of atrial fibrillation [8]. Therefore, appropriate investigation should be carried out to identify the source of the thrombus.

## 2. Case Presentation

**Figure 1:**
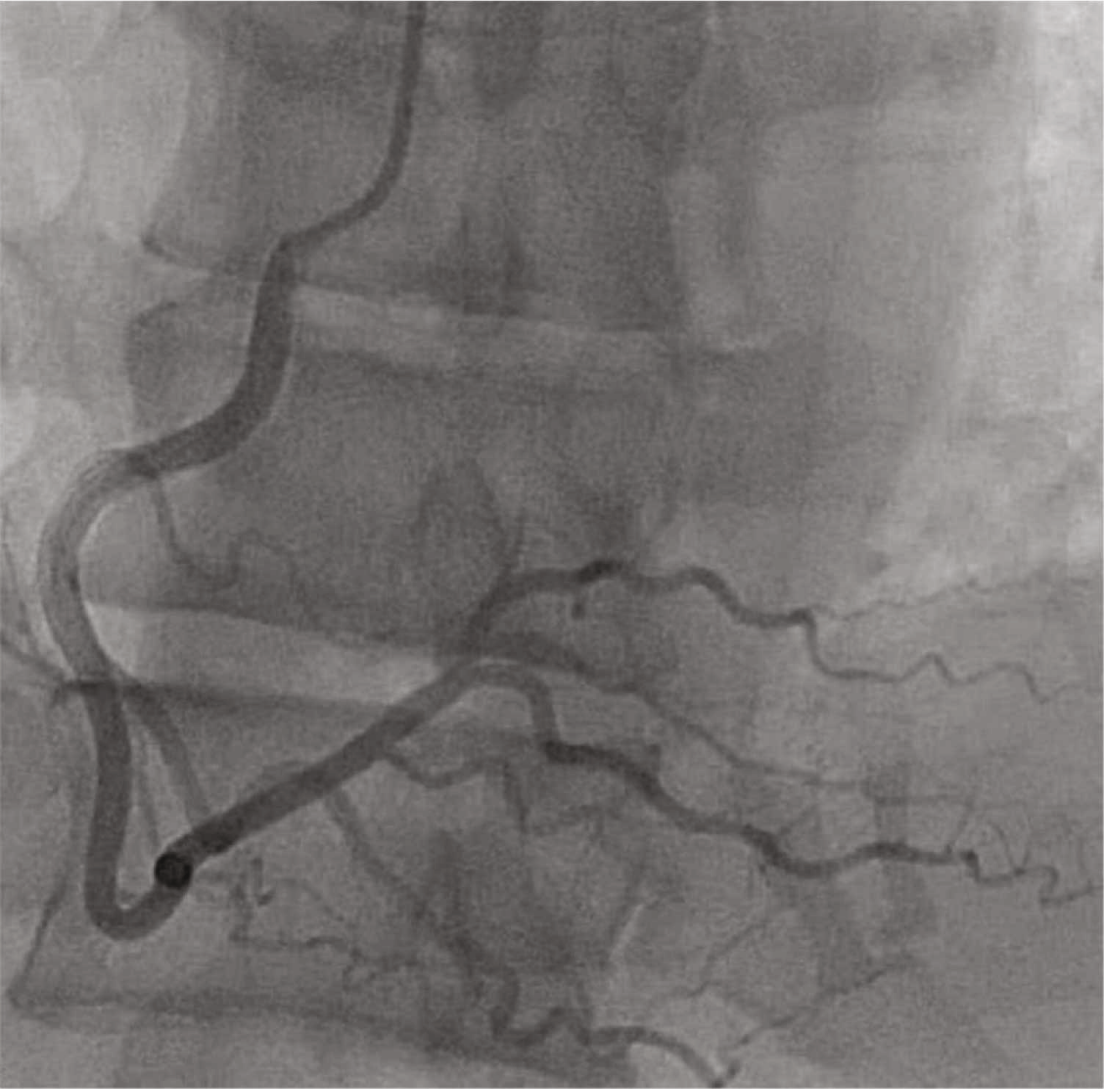
Left heart catheterization via the right radial approach. Selective angiograph of the right coronary artery in the left anterior oblique cranial projection revealing a medium caliber vessel angiographically free of any significant atherosclerotic disease.

**Figure 2:**
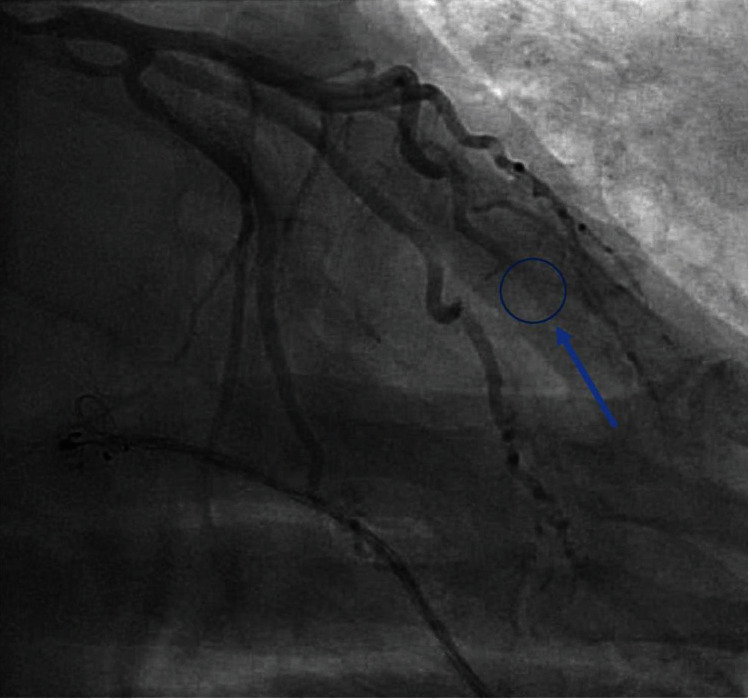
Left heart catheterization via the right radial approach. Selective angiograph of the left coronary artery in the right anterior oblique caudal projection revealing a no significant atherosclerotic disease but with a filling defect noted in the distal left anterior descending artery.

**Figure 3:**
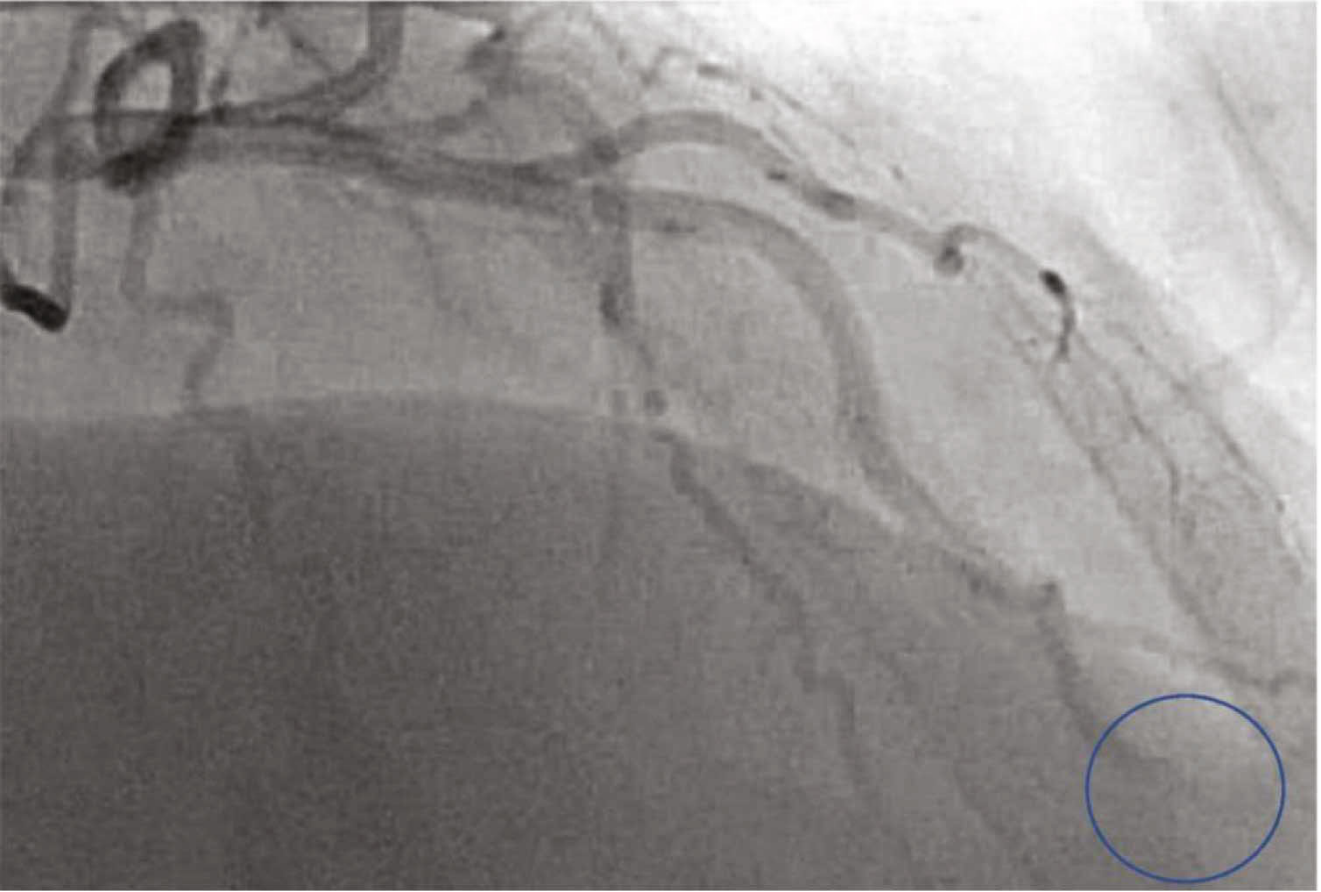
Left heart catheterization via the right radial approach. Selective angiograph of the left coronary artery in the right anterior oblique cranial projection revealing a distally occluded left anterior descending artery with thrombus (blue circle).

**Figure 4:**
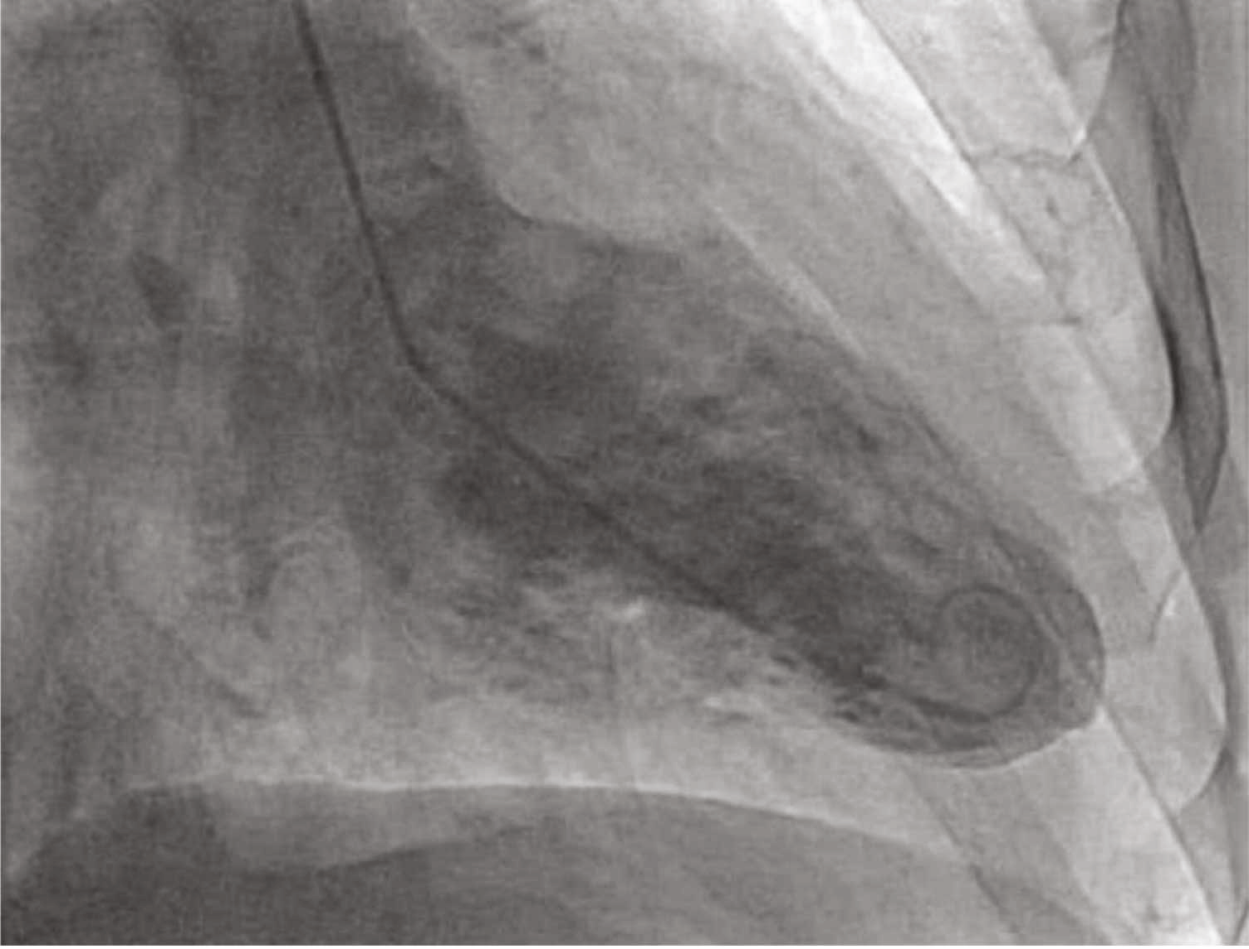
Left ventriculogram revealing a mildly reduced ejection fraction (visual estimation of 40%) along with akinesis of the distal anterior, apical anterior, and apical and apical inferior walls without evidence of an apical thrombus.

We report the case of a 53-year-old obese gentleman who presented with acute sudden onset pressure-like chest pain associated with lightheadedness. This was the first time the patient reported having chest pain. He had no exertional symptoms. He called EMS and was immediately transferred to our facility. The patient was reportedly diagnosed with paroxysmal atrial fibrillation in the past year by his primary care physician but was not placed on anticoagulation since his CHA_2_DS_2_-VASc score was 0. He had never had any stress testing done. He was otherwise a lifelong nonsmoker, and his family history was unremarkable. Vital signs were stable on admission. His physical exam was pertinent for a middle-aged gentleman in no acute distress but with an irregularly irregular rhythm, borderline tachycardic rate, and variables S1 and S2 without any murmurs, rubs, or gallops. ECG on admission revealed coarse atrial fibrillation with a rapid ventricular rate and premature ventricular contractions. Chest X-ray did not reveal any acute abnormality. Pertinent labs on admission included a normal hemoglobin and white blood cell count with mild thrombocytopenia, normal prothrombin time and activated partial thromboplastin time, and normal basic metabolic panel but with mild hyperglycemia. His initial troponin T was 0.02 ng/ml followed by 0.03 ng/ml and finally 0.80 ng/ml (0.10 ng/ml being the upper limit of normal in our lab). ProBNP was 1104 pg/ml (normal range 50–137 pg/ml). His TSH was undetectable, FT4 was 3.79 ng/dl (normal range 0.93–1.7 ng/dl), FT3 was 6.9 pg/ml (normal range 2–4.4 pg/ml), hemoglobin A1c was 5.6%, and LDL was 58 mg/dl. Transthoracic echocardiogram was done with contrast to improve opacification of the left ventricle. It revealed a mildly reduced ejection fraction (EF 45-50%), akinesis of the apical wall, normal right ventricular systolic function, mildly dilated left and right atria, mild to moderate mitral, and tricuspid valve regurgitation along with moderate pulmonary hypertension. The patient was given a full-dose aspirin, high dose statin, and a beta blocker along with a heparin drip. A left heart catheterization was performed and revealed evidence of thrombotic occlusion of the distal left anterior descending artery without any evidence of obstructive epicardial atherosclerotic coronary disease (Figures 1–4). A 6 French EBU 3.0 catheter was used to engage the left main coronary artery, and a BMW wire was used to cross the lesion, and a 2.5 mm × 16 mm balloon was used to dilate the lesion with marginal benefit likely due to organized thrombus. The patient was then started on low dose aspirin and clopidogrel for dual antiplatelet therapy. He was also started on rivaroxaban which would allow for one-time daily dosing. As well he was continued on metoprolol which was started on his presentation to the emergency room. He will continue triple therapy for one month and then continue clopidogrel and rivaroxaban. His hospitalization was otherwise uneventful and was later on discharged with close outpatient follow-up.

## 3. Discussion

Diagnosis of coronary embolism can be challenging. Our patient had no evidence of atherosclerotic disease on heart catheterization; this in the setting of atrial fibrillation greatly swayed our differential towards an embolism. However, had there been a thrombus with concomitant atherosclerotic disease, arriving at a definite diagnosis would be far more difficult. Shibata et al. have proposed criteria for diagnosis, presented in Table 1, of which our patient has met 1 major and 2 minor criteria [4]. Per their proposed method, this would suggest a definite diagnosis of coronary embolism. Nonetheless, no set standard of diagnosis has been established.

An additional challenge is to establish the etiology of the embolism. In a study conducted by Lacey et al., 147 cases of coronary embolism from 1990 to 2017 were reviewed [5]. It was found that the three most common etiologies of coronary embolism were infective endocarditis, atrial fibrillation, and prosthetic valve thrombosis, respectively. Other rare causes that have been documented include left ventricular thrombus, atrial myxoma, and papillary fibroelastoma. However, with the advancement in early diagnosis, atrial fibrillation may have surpassed infective endocarditis as the most common etiology; however, that remains a postulation as of now [5].

Thrombophilia may also be considered in patients with CE; however, the relationship between thrombophilia and arterial thromboembolism is still contested. Little data is available to link these two entities [1, 9]. The decision to pursue a thrombophilia workup should thus be made judiciously. Guiding principles may be derived from stroke management as there are too few cases of CE to establish specific guidelines. Nonetheless, this workup should only be pursued if no other obvious etiology is present, and laboratory testing will result in changes to patient management [1].

The most frequently involved vessel is the left anterior descending artery followed by the left circumflex, right coronary, and left main coronary artery, respectively [5]. This is in agreement with a study by Prizel et al., who found the left anterior descending to be the most commonly involved artery [3].

The infrequent occurrence of coronary embolism is thought to be related to the surrounding anatomy [3, 5]. With a wide aortic orifice relative to the coronary ostia, most emboli leaving the aortic outflow tract would likely continue to further organs. More so, the ostia of the coronary arteries are at sharp angles to the aorta making it further less accessible to an embolism.

Currently, there are no formal guidelines with regard to workup and management of coronary embolism [5]. Treatment must be individualized based on lesion amenability to interventional techniques as well as the underlying etiology. Lacey et al. reported use of thrombectomy in nearly half of the cases that were reviewed along with balloon angioplasty +/- stent placement along with thrombolysis. Upon discharge, a third of patients were prescribed anticoagulation +/- antiplatelet agents [5]. Shibata et al. also note that histological study may aid in diagnosis and is a benefit if the thrombus is aspirated [4]. However, a challenge remains that many emboli lodge in distal segments of culprit vessels, at times beyond what is accessible to a catheter. Such patients should continue to receive medical management. This includes anticoagulation for 6 months or 3 if a reversible risk factor has now resolved, as well as antiplatelet therapy if PCI was done [1]. Studies have not demonstrated significant mortality benefit with aspiration thrombectomy in patients with ST segment elevation myocardial infarction [10].

This case was further complicated by the patients' diagnosis of atrial fibrillation. Atrial fibrillation increases the risk of systemic thromboembolism. Interestingly, in the cohort study by Shibata et al., 18 out of 30 patients who had atrial fibrillation and developed a coronary embolism had a CHADS_2_ score of 0-1 [4]. However, when reevaluated with the CHA_2_DS_2_-VASc score, 11 of the 18 patients had a score of 2 or greater [4]. A CHA_2_DS_2_-VASc score of 2 or above is an indication for use of prophylactic anticoagulation according to the most recent guidelines published in 2019 [11]. There is uncertainty whether anticoagulation is warranted in patients with a CHA_2_DS_2_-VASc of 0 or 1; however, clinical judgment is recommended. The unadjusted stroke risk for patients with a CHA_2_DS_2_-VASc of 0, 1, and 2 is 0.2%, 0.6%, and 2.2% per year, respectively [11]. There is no evidence that treatment with aspirin for patients with low CHA_2_DS_2_-VASc scores (≤1) offers any clinical benefit [11]. Of note, hyperthyroidism was not found to be an independent risk factor for thrombosis [12, 13].

Our patient had a CHA_2_DS_2_-VASc score of 0 and absence of left ventricular and left atrial appendage thrombus on transthoracic echocardiography even though transesophageal echocardiography was not performed. Left atrial appendage occlusion devices can be considered if there is an absolute contraindication to anticoagulation or increased risk of bleeding if indeed the left atrial appendage was the source of embolism [14], but further studies are needed in this regard as the data on the use of devices for coronary embolism is limited. This case clearly illustrates the imperfection of the assumptions underlying a “low” CHA_2_DS_2_-VASc score and the importance of clinical judgment to guide the decision to anticoagulate such patients.

## 4. Conclusion

We reported a case of a 53-year-old male who was found to have coronary embolism secondary to atrial fibrillation off anticoagulation because of a CHA_2_DS_2_-VASc score of zero. Coronary embolism is rare and sometimes a challenge to diagnose. The most reported etiologies of coronary embolism are valvular masses (e.g., infective endocarditis), atrial fibrillation, and prosthetic valve thrombosis. Treatment is individualized and is based on the etiology. Even though our patient did not qualify for anticoagulation because of a low CHA_2_DS_2_-VASc score, this serves as a reminder of the imperfection of our assumptions which raises the question whether we should have a lower threshold to initiate anticoagulation. This is not to say that anticoagulation is without risks; however, a more individualized approach may be needed.

## Figures and Tables

**Table 1:** Criteria for diagnosis of coronary embolism (Shibata et al.).

Criteria for diagnosis of coronary embolism
Major criteria

Evidence of coronary embolism or thrombus angiographically without atherosclerosis.

Evidence of coronary emboli to multiple sites concomitantly.

Systemic embolization in the absence of acute myocardial infarction induced left ventricular thrombus.

Minor criteria

Stenosis of nonculprit coronary artery < 25%.

Evidence of embolic sources based on noninvasive imaging.

Presence of risk factors for emboli, cardiomyopathy, rheumatic heart disease, prosthetic valve, PFO, atrial septal defect, history of cardiac surgery, infective endocarditis, or hypercoagulable state.

Patients with 2 or more major criteria, 1 major and 2 minor, or 3 minor criteria were considered to have a definite coronary embolus. Patients with 1 major and 1 minor or 2 minor criteria were considered to have a probable coronary embolus.

## Abbreviations

CE: Coronary embolism
ACS: Acute coronary syndrome
AF: Atrial fibrillation
PFO: Patent foramen ovale
PCI: Percutaneous coronary intervention.
